# Wetland agribusiness aspects and potential in Bangladesh

**DOI:** 10.1016/j.dib.2017.11.055

**Published:** 2017-11-17

**Authors:** Aurup Ratan Dhar, Md. Monirul Islam, Arifa Jannat, Jasim Uddin Ahmed

**Affiliations:** a Department of Agricultural Economics, Bangladesh Agricultural University, Mymensingh, Bangladesh; b Institute of Agribusiness & Development Studies, Bangladesh Agricultural University, Mymensingh, Bangladesh; c Department of Agricultural Economics and Policy, Sylhet Agricultural University, Sylhet, Bangladesh

**Keywords:** Wetland, Agribusiness, Potential, Bangladesh

## Abstract

The study was conducted to document farmers’ livelihood aspects and agribusiness potentials in wetland areas of Bangladesh. A total of 120 farmers and 24 service providers were interviewed for data collection. Most of the farmers were small farmers having less than 1.0 ha of cultivable land. The differences in productivity of crop farming and poultry rearing between wetland area and main land were statistically significant. Favorable farm environment and proper utilization of agricultural resources were major strength and opportunity. The data may be helpful for formation of agribusiness clusters involving input suppliers, credit/financial organizations and different support service providers for more income, better nutrition and improved livelihood of the wetland people.

**Specifications Table**

**Table:** 

Subject area	*Agriculture, Economics, Social science*
More specific subject area	*Agribusiness*
Type of data	*Table, text file, figure*
How data was acquired	*Field survey*
Data format	*Analyzed*
Experimental factors	*Not applicable*
Experimental features	*Not applicable*
Data source location	*Mohongonj upazila of Netrakona district and Mithamoin upazila of Kishoregonj district, Bangladesh*
Data accessibility	*Data is confidential*
Related research article	*Not applicable*

**Value of the data**

• A number of studies have been conducted on economic, environmental and livelihood prospect of wetland areas in Bangladesh but there is no specific study on existing farming practices, profitability and business prospects in these areas.
• The study will be very helpful to minimize the research gap and add valuable information on the existing notions.
• The findings of the study will be used to design intervention strategies aimed at reducing the constraints to farming in the wetland region.
• The range of activities may include advocacy and awareness creation at the local and national level, promote policy changes to alleviate the business constraints.

## 1 Data

Data were collected from the sample farmers through direct interview method using a structured questionnaire. Secondary data sources were also considered. Primary data were collected from Mohongonj upazila of Netrakona district and Mithamoin upazila of Kishoregonj district. A total of 120 farmers were interviewed following stratified random sampling based on farm size. Also, a total of 24 service providers were interviewed for data collection.

## 2 Experimental design, materials, and methods

Bangladesh has witnessed respectable improvements in its economic, social and health conditions with annual GDP growth of 6.6% [1]. While the overall conditions of the country are promising, those residing in the wetland areas (locally called as ‘*haor*’) have not enjoyed the same level of relative or absolute progress [2]. The wetland areas of north-eastern region in Bangladesh cover about 2.0 million ha of area and accommodate about 19.4 million people [3]. Farming is the major economic activity of this region. The wetland region has long been lagging behind mainstream national development although the economic development of Bangladesh is moving steadily at a moderate pace. It is difficult to foresee the country's overall progress without the development of the wetland region as it covers a major part of the country and population which deserves special development initiatives. It is beyond doubt that suitable agribusiness environment will autonomously push the wetland areas to the light of development [4], [5].

SWOT analysis for business prospects and challenges of farmers is represented in Table 1. In terms of strengths, 76.7% respondents stated about favorable farm environment and 71.7% stated about enterprise interdependence; and in terms of weakness, 75.8% respondents stated about weak marketing system and 63.3% stated about lack of agricultural credit access. Considering opportunities, 72.5% respondents stated about improvement in agricultural technologies and 60.8% stated about proper utilization of agricultural resources; and in terms of threats, 84.2% respondents stated about environmental vulnerability where 80.0% stated about declining amount of cultivable land.

**Table 1:** SWOT analysis for business prospects and challenges of farmers.

Statements	% of farmers	Rank	Statements	% of farmers	Rank
Strengths			Weakness		
Increased farm productivity	53.3	3	Scarcity in input availability	57.5	3
Enterprise interdependence in the form of input-output relationship	71.7	2	High price of inputs	40.0	4
Favorable farm environment	76.7	1	Weak marketing system	75.8	1
Sufficient workforce	49.2	4	Lack of agricultural credit access	63.3	2
	
Opportunities			Threats		
Improvement in agricultural technologies	72.5	1	Price fluctuation	64.2	4
Diversification in farming practices	57.5	3	Environmental vulnerability	84.2	1
Proper utilization of agricultural resources	60.8	2	Declining amount of cultivable land	80.0	2
Market potential for value added agricultural products	45.0	4	Land transformation from cropland to others	68.3	3

**Fig. 1:**
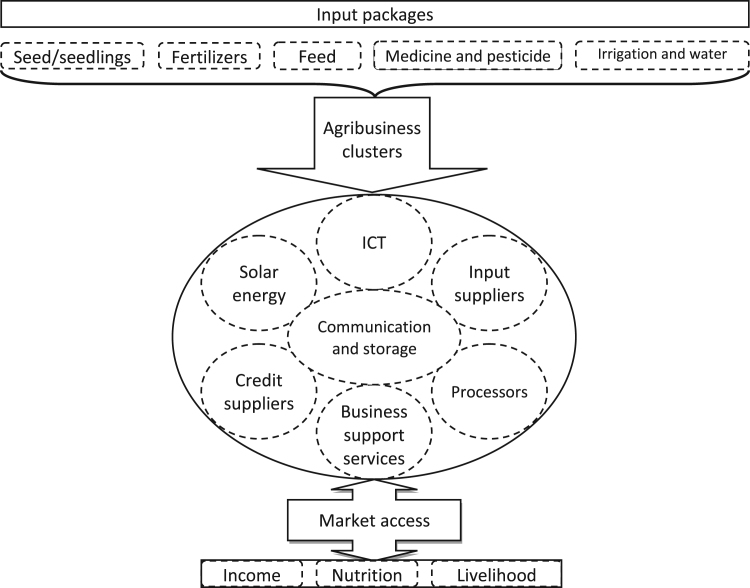
Conceptual framework of agribusiness perspectives in wetland areas.

The data demand to develop some forms of agribusiness activities based on local agriculture and rural community which ultimately contribute for the betterment of wetland economy, farmers’ income generation and livelihood improvement. Fig. 1 depicts that targeting on crucial agricultural input packages, agribusiness clusters with the involvement of input suppliers, credit/financial organizations, different support service providers may be formed. Such supportive agribusiness environment will contribute notably for farmers’ employment creation and income generation. Thereby, farmers’ market access will be easier through more income yielding from different agricultural enterprises, better nutrition will be ensured and livelihood of the wetland people will be improved.

The study areas hold a high agribusiness environment with plenty of local resources. Higher local and regional demand for good quality agricultural energy inputs (i.e., fertilizers, pesticides, feed, etc.) has created possible opportunities for fertilizers and pesticides industries, feed mills, etc. Now-a-days, modern agricultural equipments and machineries create crying need to these areas. Increased rice productivity and large amount of fish availability in the wetlands show great prospects of establishing rice mills and fish processing industries (Table 2).

**Table 2:** Available resources and extent of agribusiness opportunity.

Enterprises involved	Avenue of agribusiness potential	Available resources	Considerable issues	Extent of opportunity based on researchers’ observation		
				High	Medium	Low
Crop	Fertilizers and pesticides industries	Labour abundance and availability of quality ingredients	Higher demand and market price, and employment opportunities		√	
	Seed processing industries	Availability of high quality grains	Lack quality seed and demand for high yielding variety seeds			√
	Rice mills	Higher rice productivity and labour availability	Risk of paddy damage and immediate course of action, and higher demand for processed grains	√		
Livestock and poultry	Feed mills	Availability of quality ingredients	Demand for quality feed; and lack of specialized feed for each of livestock and poultry		√	
Fish capture	Fish processing industries	Plenty of different kinds of fishes	Higher demand of processed fish and export potential	√		
All agricultural enterprises	Agricultural equipment and machinery industries	Availability of manpower, and equipment producing and processing elements	Lack of necessary agricultural equipments and machineries in time, and higher demand and market price	√		
	Transportation vehicles and storage	Availability of labour and scope of investment	Higher demand due to product perishability	√		

## References

[1] WB, GDP Growth (Annual %), , (2016). World Bank National Accounts Data.

[2] Sharma P.K. (2010). Scenario of haor vulnerabilities and other obstacles for sustainable livelihood development in Nikli upazila. J. Bangladesh Agric. Univ..

[3] MoWR, Master plan of *haor* area, Bangladesh *Haor* and Wetland Development Board, Ministry of Water Resources, Government of the People’s Republic of Bangladesh, vol. 1, 2012, pp. 1–55.

[4] Khan M.N.H., Mia M.Y., Hossain M.R. (2012). Impacts of flood on crop production in haor areas of two upazillas in Kishoregonj. J. Environ. Sci. Nat. Resour..

[5] Islam S., Uddin M.T., Akteruzzaman M. (2011). Profitability of alternate farming systems in Dingapota haor area of Netrokona district. Prog. Agric..

